# Evaluating the impact of digital therapy for male LUTS: insights from the BEST trial subgroup analysis

**DOI:** 10.1007/s00345-025-06016-2

**Published:** 2025-11-01

**Authors:** Laura Wiemer, Christian Gratzke, Kurt Miller, Erik Krieger, C. Patrick Papp, Sandra Schönburg

**Affiliations:** 1 Department of Urology, Charité, Universitätsmedizin Berlin, Berlin, Germany; 2 Department of Clinical Research, Kranus Health GmbH, Munich, Germany; 3 Department of Urology, University Medical Center Freiburg, Freiburg im Breisgau, Germany; 4 BG Hospital Bergmannstrost, Halle (Saale), Germany; 5 Department of Urology, University Hospital Halle (Saale), Halle (Saale), Germany

**Keywords:** Digital therapy, Male LUTS, BPH, OAB, Subgroups

## Abstract

**Purpose:**

Men with lower urinary tract symptoms (LUTS) represent a heterogeneous patient population. The BEST trial investigated the effectiveness of a digital health application for male LUTS. This pre-specified subgroup analysis evaluated treatment effects based on key clinical characteristics and additionally reports micturition diary outcomes and adherence data.

**Methods:**

In this randomized controlled trial, 237 patients were assigned to either the intervention group (IG, *n* = 112), receiving app-based therapy (Kranus Lutera) alongside standard care, or the control group (CG, *n* = 125), receiving standard care alone. Primary endpoints included 12-week changes in the International Prostate Symptom Score (IPSS) and both subscales of the Overactive Bladder Questionnaire Short Form (OAB-q SF). Subgroup analyses were stratified by diagnosis (OAB, BPH, OAB + BPH), baseline symptom severity, age (≤ 60, > 60 years), and concurrent pharmacological treatment. Additional outcomes included changes in daytime/nighttime voiding frequency and urgency episodes. German Clinical Trials Registry number: DRKS00030935.

**Results:**

Subgroup analyses showed consistent improvements after 12 weeks in favour of the IG across diagnoses (between-group differences IPSS − 6.4 to -7.4), with the largest improvement in severe LUTS (-10.7; 95% CI -12.7;-8.6, *p* < 0.0001). Patients benefited regardless of age or medication use. These findings were further supported by improvements in OAB scores. Micturition diaries showed reduced daytime (-1.33), nighttime (-0.18), and urgency episodes (-1.59). In the IG, 84% of participants used the app several times per week.

**Conclusion:**

The digital therapeutic achieved clinically meaningful symptom and quality-of-life improvements across all subgroups. Additional reductions in voiding frequency and high adherence support its broad applicability in clinical practice.

## Background

Digital health interventions are increasingly integrated into routine care to support disease detection, symptom monitoring, and therapeutic management across a wide range of medical disciplines. In Germany, for example, certified digital health applications are regulated as low-risk medical devices under the Medical Device Regulation (MDR class I–IIb) and are reimbursable within the statutory health insurance system [1]. Similar frameworks exist internationally, reflecting a growing recognition of their potential to complement or replace elements of traditional care pathways [2, 3]. Lower urinary tract symptoms (LUTS) are highly prevalent among men and can significantly impact quality of life [4, 5]. In the overall cohort of the Bladder Emptying DiSorder Therapy (BEST) study, the digital health application Kranus Lutera demonstrated significant clinical benefits. Specifically, participants experienced improvements in voiding symptoms as measured by the International Prostate Symptom Score (IPSS; between-group difference: -7.0 points; 95% CI: -8.1 to -5.9; *p* < 0.0001), a reduction in symptom burden according to part 1 of the Overactive Bladder questionnaire short form (OAB-q SF; between-group difference: -18.6 points; 95% CI: -22.2 to -15.0; *p* < 0.0001), and an increase in health-related quality of life (HRQOL) as measured by part 2 of the OAB-q SF (between-group difference: +17.2 points; 95% CI: 14.18 to 20.16; *p* < 0.0001) [6].

To further assess the robustness of this treatment and identify potential effect modifiers, we now present data from the pre-specified subgroup analyses examining whether treatment effects differ by diagnosis, baseline symptom severity, age, or concomitant medication use, but also analyses of the micturition diaries used within the app. These analyses aim to determine whether specific patient characteristics are associated with differential treatment benefit, thereby supporting a more personalized approach to digital therapy in male LUTS management.

## Materials and methods

The BEST study was a two-arm, randomized, controlled, bicentric, single-blind study assessing Kranus Lutera as treatment for male LUTS [6]. The present report focuses on pre-specified subgroup analyses and additional micturition diary data, all defined in the statistical analysis plan prior to unblinding.

Participants were initially diagnosed and recruited by office-based urologists across Germany as part of routine clinical practice. These urologists assigned patients to diagnostic categories of overactive bladder (OAB), benign prostatic hyperplasia (BPH), or a combination of both (OAB + BPH) based on their clinical judgment. Subsequently, study center physicians verified the diagnoses to ensure consistency across sites. To standardize inclusion and confirm symptom burden, validated questionnaires were administered. Eligibility criteria included an International Prostate Symptom Score (IPSS) of ≥ 4 and/or an Overactive Bladder Questionnaire Short Form (OAB-q SF) Part 1 score of ≥ 18. This combined approach of clinical diagnosis and symptom-based validation ensured robust and reproducible subgroup classification. The study was not powered to detect interaction effects between treatment and subgroups. As such, any potential differences in treatment response across subgroups should be interpreted as exploratory and hypothesis-generating.

Participants in the intervention group (IG) received standard care plus access to the digital therapy Kranus Lutera, while the control group (CG) received standard care alone and was given access to the therapy after 12 weeks. No sham application was used.

The app delivers a structured 12-week program that integrates evidence-based modules combining physical, psychological, and behavioral therapy components [6]. These include pelvic floor muscle training, bladder training, fluid management, behavioral and urge control strategies, and the use of a digital micturition diary. The program is delivered through a multimedia interface that includes video demonstrations, audio instructions, haptic feedback elements, and written educational content. Users are guided through daily exercises and educational sessions that adapt dynamically based on individual progress. Motivational features such as progress tracking, reminders, and individualized feedback are integrated to support long-term engagement and adherence. The content and structure of the app are based on current guidelines for the conservative management of LUTS and reflect a multimodal therapeutic approach.

The primary endpoint was the change from baseline in IPSS score. Secondary endpoints included changes from baseline in OAB-q SF parts 1 (LUTS) and 2 (quality of life).

Micturition diary data were collected in a 3-day in-app diary to assess changes in daytime and nighttime voiding frequency and episodes of urgency. Adherence to the app-based intervention was monitored both by participant self-report and via backend app usage logs. “Active use” was only tracked if the completion of the activity, task, or exercise was finalized within the app.

All statistical analyses were conducted using SAS^®^ software (Version 9.4). Statistical significance was set at 0.05 (two-sided).

The primary efficacy analyses were conducted within the intention-to-treat (ITT) population. Confirmatory endpoints were tested sequentially according to a predefined order and family-wise significance level (α = 0.05) as previously described [6]. All other endpoints were considered exploratory and are presented without adjustment for multiple comparisons.

Group differences in mean changes were assessed using ANCOVA, with treatment group as a factor and baseline values as the only covariate. Type III sums of squares were applied. No formal statistical tests for subgroup-by-treatment interaction were performed. Least squares means (LS means) were calculated for between-group comparisons.

Missing data were handled using a conservative Jump-to-Reference (J2R) imputation strategy, referencing the control group. Data on bladder metrics were collected via the app’s optional diary feature and analyzed within the per-protocol (PP) population, without imputation of missing values.

## Results

All diagnostic subgroups (Table 1) showed significant improvements, with the greatest benefits observed in patients with urge-related symptoms (LS mean difference: -7.3 points [OAB] vs. -6.4 points [BPH] vs. -7.4 points [OAB + BPH]). Correspondingly, significant improvements were also observed in OAB-q-SF scores (-20.2 [OAB] vs. -15.5 [BPH] vs. -22.1 [OAB + BPH]) and HRQOL scores (17.2 [OAB] vs. 14.8 [BPH] vs. 20.6 [OAB + BPH]).

In terms of baseline symptom severity (Table 1), patients with severe LUTS (IPSS ≥ 20 points) experienced the most pronounced improvements (LS mean difference: -10.7 points vs. -4.6 points [IPSS 8–19] vs. -10.1 points [IPSS 0–7]). OAB-q-SF scores similarly improved with increasing severity (-25.4 [severe] vs. -14.9 [moderate] vs. -17.5 [mild]), with corresponding HRQOL gains (25.5 [severe] vs. 12.8 [moderate] vs. 7.8 [mild], though the latter was not statistically significant [*p* = 0.2148]).

Across age groups (Table 1), older patients benefited to a comparable extent (LS mean difference: -7.6 points [≤ 60 years] vs. -6.3 points [> 60 years]), with associated improvements in OAB-q-SF (-18.0 vs. -19.6) and HRQOL (18.1 vs. 16.1). The maximum age observed among participants was 88 years. In the intervention group, 13% of individuals were aged over 70 years. Patients receiving concurrent pharmacological treatment for OAB/BPH (Table 1) also showed significant symptom improvement (LS mean difference: -5.5 points [with medication] vs. -7.3 points [without medication]). The OAB-q-SF and HRQOL outcomes supported these findings (-16.6 and 14.6 [with medication] vs. -19.0 and 18.1 [without medication]). Among those using medication, approximately 40% received alpha-blockers, 30% antimuscarinics, and the rest were treated with beta-3 mimetics, PDE-5 inhibitors, 5-alpha-reductase inhibitors, or phytotherapy. Thus, a significant treatment effect was also evident among those already receiving medication.

Patients with higher BMI (> 25 kg/m²) showed comparable improvements compared to those with lower BMI (IPSS − 7.2 vs. -6.5, OAB-q-SF: -18.2 vs. -18.5, HRQOL: 17.1 vs. 17.5).

No substantial differences were found between study centers in subgroup analysis (IPSS − 7.6 vs. -6.6, OAB-q SF Part 1 -17.3 vs. -18.7, OAB-q SF Part 2 -11.2 vs. -11.2).

**Fig. 1:**
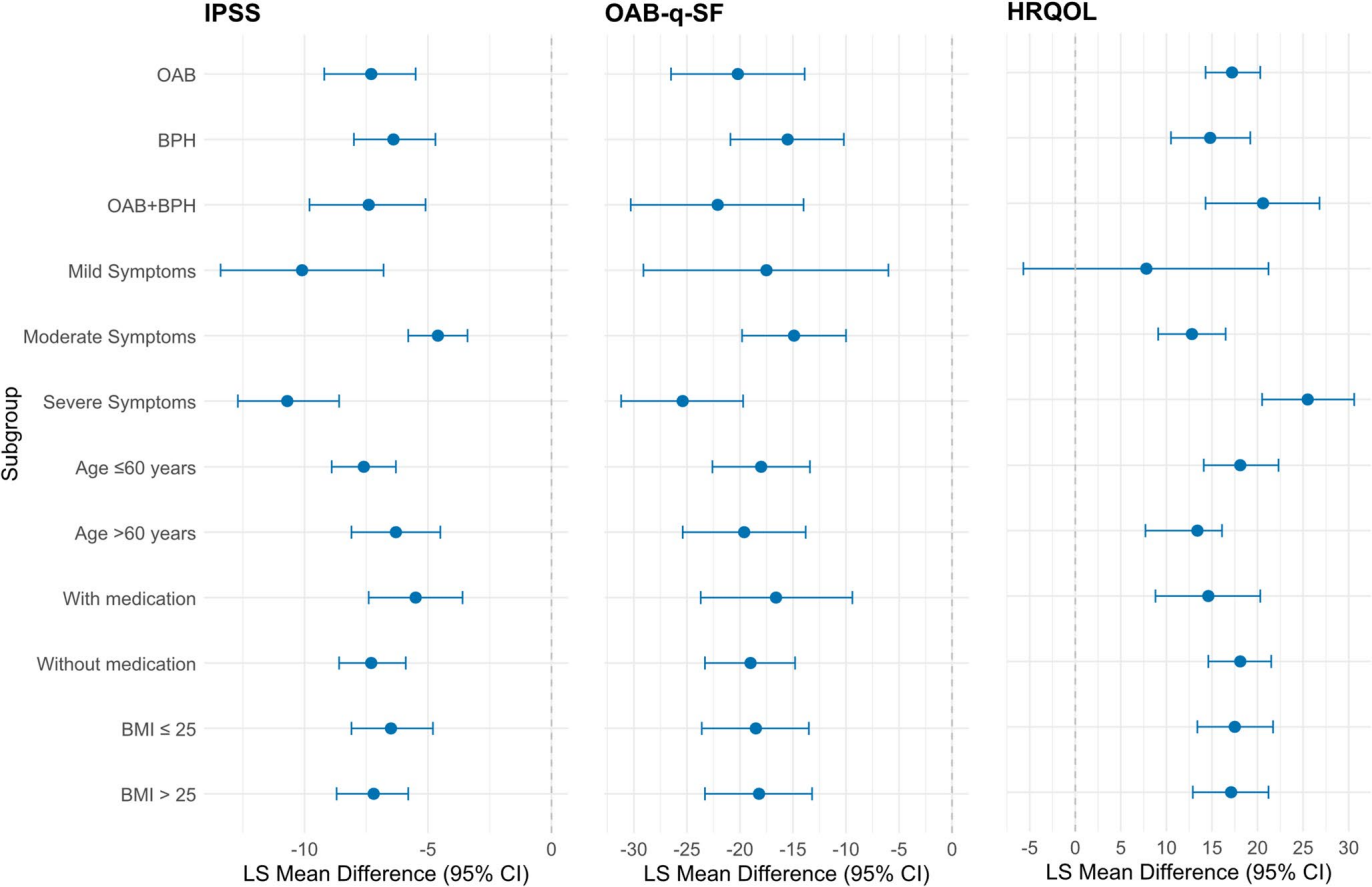
Forest Plot of LS Mean Differences by Subgroup and Outcome

In summary, the intervention demonstrated robust and statistically significant efficacy across all subgroups. The greatest effects were observed in patients with more severe symptoms and those with urge incontinence components. Symptom improvement was independent of age and concurrent medication. These subgroup findings for all three outcome measures are summarized in Fig. 1.

Among the 106 participants in the app group (ITT population), 95 (90%) provided data on daytime micturition frequency and urgency episodes at baseline, and 72 (68%) at 12 weeks. For nighttime frequency, 85 participants (80%) submitted data at baseline and 55 (52%) at follow-up. Among these participants, daytime voiding frequency decreased by 1.3 episodes, nighttime frequency by 0.2, and urgency episodes by 1.6 between baseline and week 12. Furthermore, high treatment adherence was observed in the intervention group, with 84% of participants reporting app use several times per week. Objective app data showed that 83% of participants were still active in the final week of the intervention, with a consistent activity rate of approximately 83% in each preceding week. Self-reported adherence differed by only one participant, who may have performed exercises outside the app interface. The close agreement between self-report and backend logs supports the validity of the high adherence rate.

**Table 1:** Changes of the IPSS, OAB-q-SF, and HRQOL scores for relevant LUTS subgroups – ITT

Subgroup	Sample size		IPSS			OAB-q-SF			HRQOL		
			LS Mean Difference	95% CI	*p* Value	LS Mean Difference	95% CI	*p* Value	LS Mean Difference	95% CI	*p* Value
	IG	CG									
**OAB** **(N32.8)**	**32**	**42**	**-7.3**	**-9.2;-5.5**	**< 0.0001**	**-20.2**	**-26.5;-13.9**	**< 0.0001**	**17.2**	**14.3; 20.3**	**< 0.0001**
**BPH** **(N40)**	**54**	**56**	**-6.4**	**-8.0;-4.7**	**< 0.0001**	**-15.5**	**-20.9;-10.2**	**< 0.0001**	**14.8**	**10.5; 19.2**	**< 0.0001**
**OAB + BPH** **(N32.8 + N40)**	**26**	**27**	**-7.4**	**-9.8;-5**,**1**	**< 0.0001**	**-22.1**	**-30.3;-14.0**	**< 0.0001**	**20.6**	**14.3; 26.8**	**< 0.0001**
**Mild Symptoms** **(0–7 IPSS points)**	**6**	**4**	**-10.1**	**-13.4;-6.8**	**0.0002**	**-17.5**	**-29.1;-6.0**	**0.0088**	**7.8**	**-5.7; 21.2**	**0.2148**
**Moderate Symptoms** **(8–19 IPSS points)**	**68**	**71**	**-4.6**	**-5.8;-3.4**	**< 0.0001**	**-14.9**	**-19.8;-10.0**	**< 0.0001**	**12.8**	**9.1; 16.5**	**< 0.0001**
**Severe Symptoms** **(20–35 IPSS points)**	**38**	**50**	**-10.7**	**-12.7;-8.6**	**< 0.0001**	**-25.4**	**-31.2;-19.7**	**< 0.0001**	**25.5**	**20.5; 30.6**	**< 0.0001**
**Age ≤ 60 years**	**56**	**71**	**-7.6**	**-8.9;-6.3**	**< 0.0001**	**-18.0**	**-22.6;-13.4**	**< 0.0001**	**18.1**	**14.1; 22.3**	**< 0.0001**
**Age > 60 years**	**56**	**54**	**-6.3**	**-8.1;-4.5**	**< 0.0001**	**-19.6**	**-25.4;-13.8**	**< 0.0001**	**13.4**	**7.7; 16.1**	**< 0.0001**
**With medication for OAB/BPH**	**31**	**38**	**-5.5**	**-7.4;-3.6**	**< 0.0001**	**-16.6**	**-23.7**,**-9.4**	**< 0.0001**	**14.6**	**8.8; 20.3**	**< 0.0001**
**Without medication for OAB/BPH**	**81**	**87**	**-7.3**	**-8.6;-5.9**	**< 0.0001**	**-19.0**	**-23.3;-14.8**	**< 0.0001**	**18.1**	**14.6; 21.5**	**< 0.0001**
**BMI ≤ 25 kg/m** ^**2**^	**42**	**52**	**-6.5**	**-8.1;-4.8**	**< 0.0001**	**-18.5**	**-23.6**,**-13.5**	**< 0.0001**	**17.5**	**13.4; 21.7**	**< 0.0001**
**BMI > 25 kg/m** ^**2**^	**70**	**73**	**-7.2**	**-8.7;-5.8**	**< 0.0001**	**-18.2**	**-23.3**,**-13.2**	**< 0.0001**	**17.1**	**12.9; 21.2**	**< 0.0001**

## Discussion

This exploratory analysis of pre-specified subgroups from the BEST trial suggests that digital therapy for men with LUTS can achieve consistent and clinically meaningful improvements in both lower urinary tract symptoms and health-related quality of life, irrespective of diagnostic category (BPH, OAB, or both), baseline symptom severity, age, BMI, or concomitant medication use. All subgroups achieved mean changes exceeding the MCID reported in the literature for both IPSS (3 to 5 points) and OAB-q SF (10 points) [7–9]. Patient preferences for conservative management of LUTS are known to vary according to symptom severity and perceptions of treatment risk and benefit, with men experiencing milder symptoms often opting for conservative or lower-risk approaches [10]. Evidence from primary care trials indicates that standardized, manualized conservative interventions can achieve sustained improvements without increasing adverse events or referral rates [11]. Our findings suggest benefits across all severity strata, with the largest absolute gains in patients with severe baseline symptoms (IPSS ≥ 20), who also showed the largest HRQOL improvements (25.5%). This aligns with broader observations that greater baseline symptom burden is associated with larger absolute improvements [7, 8], and supports the relevance of conservative therapy even in patients often considered candidates for invasive or pharmacological treatments. Thus, conservative care may have utility beyond those with mild disease, potentially delaying or reducing the need for escalation. However, these observations are descriptive only; no formal interaction testing was performed.

The benefits of conservative self-management interventions for LUTS are further supported by evidence from a meta-analysis demonstrating a clinically important reduction in IPSS at 6 months compared with usual care (mean difference [MD] = − 7.4; 95% CI, − 8.8 to − 6.1) [12]. This symptom improvement is comparable to that achieved with pharmacological therapies at 6 to 12 weeks (MD = 0.0; 95% CI, − 2.0 to 2.0; three studies), indicating that self-management offers an effective alternative treatment modality. Moreover, self-management provided an additional modest benefit when combined with drug therapy at 6 weeks (MD = − 2.3; 95% CI, − 4.1 to − 0.5; one study), suggesting its role as an adjunctive strategy. The benefit observed in our subgroup analysis in patients receiving concurrent LUTS-specific medication (alpha-blockers, antimuscarinics, or beta-3 agonists) suggests that this digital intervention may serve as a valuable adjunctive tool, potentially enhancing or complementing pharmacological effects. Conversely, patients not on pharmacotherapy also experienced significant improvements, indicating the utility of the digital approach as a standalone option. This may be especially relevant for patients with contraindications to medication, concerns about adverse effects, or a preference for non-pharmacologic management strategies. Furthermore, qualitative interviews in the NHS trial showed men valued structured self-management, but this is insufficiently embedded in general practitioner consultations [11]. Digital delivery may address current gaps in conservative LUTS care by embedding structured self-management in a format that is scalable and less dependent on general practitioner consultation time.

Notably, the magnitude of improvement observed in our analysis exceeded that reported in primary care delivery trials, which may reflect higher engagement and adherence facilitated by the digital format. The low drop-out rate of seven likely also reflects the high symptom burden and motivation of participants in addressing a sensitive issue but also highlights the importance of patient-centric digital therapy design. These findings are further supported by a recent post-hoc analysis of the BEST trial, which showed that the digital intervention produced improvements across all IPSS items, with particularly pronounced effects on storage symptoms, suggesting that structured app-based therapy may be especially effective in this symptom domain [13].

Of particular note, comparable efficacy was observed in patients aged over 60 years. Digital health applications are often assumed to have limited utility in older adults due to concerns about digital literacy. However, in the app group, 13% of participants were aged over 70 years, with the oldest reaching 88 years, challenging assumptions about limited digital literacy in older adults. A potential source of selection bias must be acknowledged, as access to the application required ownership of a smartphone or tablet. Individuals unfamiliar with such technology were thus inherently excluded from participation.

In addition to improvements in IPSS and OAB-q SF scores, the digital intervention led to favourable changes in objective voiding behaviour, as demonstrated by micturition diary data. Both total daytime voiding frequency and the number of daily urgency episodes decreased by approximately one episode per day following the digital intervention. These improvements complement the questionnaire-based outcomes and may indicate behavioral changes in daily life. Guidelines recommend behavioral modification as a first-line or concurrent therapy for men with LUTS, emphasizing individualized patient education and support [4]. Such benefits are dependent on consistent adherence and successful integration of conservative strategies into daily routines. In the present study, adherence was high, with 84% of participants in the intervention group reporting app use several times per week which was confirmed by backend app data. This high engagement level suggests that the program was feasible and well integrated into patients’ daily routines - an important consideration for real-world effectiveness. Nevertheless, long-term adherence requires further evaluation.

Several strengths of this study merit emphasis. The subgroup analyses were pre-specified in the study protocol and conducted within the context of a randomized controlled trial, enhancing internal validity. The use of validated and disease-specific outcome measures (IPSS and OAB-q SF) ensures clinical relevance.

However, certain limitations should be acknowledged. Although pre-specified, the subgroup analyses were not powered for interaction testing; therefore formal statistical comparisons between subgroups should be interpreted with caution and regarded as exploratory. Furthermore, the 12-week follow-up period does not permit conclusions about long-term adherence, sustainability of symptom improvement, or prevention of disease progression. However, the longer-term follow-up assessment is ongoing. Several studies on self-management programs for LUTS assess endpoints at longer follow-up periods (6 or 12 months), often reporting further symptom improvement over time [12]. Furthermore, due to the nature of the intervention, blinding of participants was not feasible, which may have introduced expectancy bias, particularly given the reliance on subjective outcome measures such as IPSS and OAB-q SF. Additionally, a sham application was not used in the control group, which presents a potential bias, as the use of a generic health app could itself yield beneficial effects. Regarding severity subgroups, due to the smaller sample size in the subgroup with mild symptoms, confidence intervals were comparatively wide, limiting precision. This underrepresentation is common in LUTS research, as patients with bothersome or moderate-to-severe LUTS are more likely to seek and require intervention, thus, many trials and meta-analyses,particularly those assessing cardiovascular risk or treatment efficacy,compare men with moderate to severe symptoms to those with mild or no symptoms, sometimes grouping the latter together [14].

While only 13% of participants were aged ≥ 70 years, likely reflecting barriers in digital literacy, half of the patients were over 60. In this over-60 subgroup, we observed significant and clinically meaningful improvements in symptom scores and in HRQOL. Although the ≥ 70 subgroup remains small, the improvements seen among older users indicate that, once enrolled, they too can benefit meaningfully from app-based therapy. Regarding data on micturition frequency and urgency episodes, these were only documented if participants chose to use the app’s diary. Consequently, it is unclear whether the lack of data is due to an absence of symptoms or due to no entries. Given the optional nature of diary completion, these findings are based on a subset of the population and may be subject to selection bias. Participants who completed the diary may have been more engaged or symptomatic, which limits the generalizability of these outcomes to the full ITT population.

In real-world clinical practice, where pharmacological treatments are often quickly initiated or patients are simply observed (watchful waiting) [15], a digital therapy, including monitoring, lifestyle counseling, pelvic floor training, nutrition, mental health, and education on the underlying condition, can help bridge a care gap for a broad spectrum of patients with LUTS. From a clinical perspective, these exploratory subgroup findings suggest the potential of this digital intervention as a versatile tool within the therapeutic algorithm for male LUTS. Nevertheless, these results stem from a 12-week randomized trial and should be interpreted accordingly. While high adherence and symptom improvements under trial conditions are encouraging, the long-term effectiveness and applicability in real-world settings remain to be confirmed. Ongoing long-term follow-up and upcoming implementation studies will help address these important questions.

## Conclusions

This exploratory subgroup analysis suggests that the digital intervention may be beneficial across a range of patient characteristics, including age, diagnosis (OAB vs. BPH), symptom severity, and medication use. Improvements in symptom burden and health-related quality of life were observed, with the most pronounced effects in patients with more severe baseline symptoms. However, findings related to the mild symptom subgroup should be interpreted with caution due to the small sample size and longer-term follow-up will be essential to determine the durability of these effects. Nevertheless, these results support the potential utility of app-based therapy as a conservative and accessible option for men with LUTS, either alone or as part of a multimodal treatment strategy.

## Data Availability

Data is provided within the manuscript or supplementary information files.
